# Fat distribution and longitudinal anthropometric changes in HIV-infected men with and without clinical evidence of lipodystrophy and HIV-uninfected controls: A substudy of the Multicenter AIDS Cohort Study

**DOI:** 10.1186/1742-6405-6-8

**Published:** 2009-05-13

**Authors:** Todd T Brown, Xiaoqiang Xu, Majnu John, Jaya Singh, Lawrence A Kingsley, Frank J Palella, Mallory D Witt, Joseph B Margolick, Adrian S Dobs

**Affiliations:** 1 Johns Hopkins University, Baltimore, MD, USA; 2 Children's Hospital of Philadelphia, Philadelphia, PA, USA; 3 Saint Clare's Hospital, Dover, NJ, USA; 4 University of Pittsburgh, Pittsburgh, PA, USA; 5 Northwestern University Feinberg School of Medicine, Chicago, IL, USA; 6 David Geffen School of Medicine at UCLA and Harbor-UCLA Medical Center, Los Angeles, CA, USA

## Abstract

**Background:**

Fat abnormalities are common among HIV-infected persons, but few studies have compared regional body fat distribution, including visceral fat, in HIV-infected and HIV-uninfected persons and their subsequent trajectories in body composition over time.

**Methods:**

Between 1999 and 2002, 33 men with clinical evidence of lipodystrophy (LIPO+), 23 HIV-infected men without clinical evidence of lipodytrophy (LIPO-), and 33 HIV-uninfected men were recruited from the four sites of the Multicenter AIDS Cohort Study (MACS). Participants underwent dual-energy x-ray absorptiometry, quantitative computerized tomography of the abdomen and thigh, and circumference measurements of the waist, hip and thigh. Circumference measurements at each semi-annual MACS visit between recruitment and 2008 were used to compare average annual anthropometric changes in the 3 groups.

**Results:**

Body mass index (BMI) was lower in LIPO+ men than in the LIPO- men and the HIV- uninfected controls (BMI: 23.6 ± 0.4 vs 26.8 ± 1.5 vs 28.7 ± 0.9 kg/m^2^, respectively, p < 0.001). The average amount of visceral adipose tissue (VAT) was similar in all three groups (p = 0.26), but after adjustment for BMI, VAT was higher in the LIPO+ group (169 ± 10 cm^2^) compared to the LIPO- men (129 ± 12 cm^2^, p = 0.03) and the HIV-uninfected group (133 ± 11 cm^2^, p = 0.07). Subcutaneous adipose tissue (thigh, abdomen) and total extremity fat were less in the HIV-infected men (LIPO+ and LIPO-) than in the HIV-uninfected men. Over an average of 6 years of follow-up, waist circumference increased at a faster rate in LIPO+ group, compared to the LIPO- men (0.51 cm/year vs 0.08 cm/year, p = 0.02) and HIV-uninfected control men (0.21 cm/year, p = 0.06). The annual changes in hip and thigh circumferences were similar in all three groups

**Conclusion:**

Subcutaneous lipoatrophy was observed in HIV-infected patients, even those without clinical evidence of lipodystrophy, compared to age-matched HIV-uninfected men. Despite markedly lower BMI, HIV-infected men with lipodystrophy had a similar amount of VAT as HIV-uninfected men and tended to have more rapid increases in waist circumference over 6 years of follow-up. These longitudinal increases in waist circumference may contribute to the development of cardiovascular risk in HIV-infected patients with lipodystrophy.

## Introduction

In the era of highly active antiretroviral therapy (HAART), body habitus changes occur frequently among HIV-infected patients[1]. These include lipohypertrophy of the visceral compartment, breasts, and the upper back (dorsocervical fat pad) and subcutaneous lipoatrophy of the trunk, face and extremities. Studies of risk factors for the development of lipohypertrophy and lipoatrophy have generally evaluated only HIV-infected patients[2]. However, in order to ascertain the uniqueness and relative clinical importance of body composition changes that occur among HIV-infected patients, comparison to a HIV-uninfected control group is essential. Cross-sectional studies assessing cardiometabolic risk in HIV-infected patients have demonstrated greater waist circumferences[3] and waist:hip ratios [4], but smaller hip and thigh circumferences[3], compared to well-characterized HIV-uninfected control populations.

Relatively few studies, however, have compared fat distribution in HIV-infected and HIV-uninfected individuals using techniques that can separate subcutaneous and visceral fat in the abdomen, such as quantitative computerized tomography (CT) or magnetic resonance imaging (MRI). The largest study to date that has compared body composition in HIV-infected men and women to HIV-uninfected controls is the Study of Fat Redistribution and Metabolic Change in HIV Infection (FRAM). Data from this study indicated that HIV-infected men and women with clinical lipoatrophy had less visceral adipose tissue (VAT) than HIV-uninfected controls[5,6]. Another large cross-sectional study also described less peripheral fat, but more VAT, in HIV-infected versus HIV-uninfected women, despite similar average body mass indices (BMI) in both groups[7].

Even fewer studies have compared longitudinal changes in body composition in HIV-infected and HIV-uninfected individuals. In the Multicenter AIDS Cohort Study (MACS), we found that waist circumference increased more rapidly in HIV-infected men compared to HIV-uninfected men after adjustment for cumulative antiretroviral exposure, although baseline waist circumference was markedly lower in HIV-infected men[8]. This more rapid increase in waist circumference associated with HIV-infection may represent a "return to health" phenomenon, whereby effective antiretroviral therapy allows a return to the pre-morbid body composition and "catch-up" to HIV-negative peers.

We conducted a substudy in the MACS whose primary goal was to compare fat distribution, including VAT, in HIV-infected men with and without lipodystrophy to HIV-uninfected men using direct quantitative measurements, in addition to anthropomorphic measurements. Furthermore, to better understand body shape changes over time, we examined the relationship between these data and longitudinal changes in anthropometry in these three groups over the 6 years following the cross-sectional assessment.

## Methods

### Study Population

The MACS is an ongoing multicenter (Pittsburgh, PA; Baltimore, MD; Chicago, IL and Los Angeles, CA) prospective cohort study of homosexual and bisexual men who are followed on a semi-annual basis. Each semi-annual MACS visit includes a detailed medical history, a physical examination, and collection of biological specimens. The institutional review boards at each site approved study protocols and forms, and each participant provided written informed consent both for the overall study and this substudy.

### Sampling methods for the Lipodystrophy Substudy

Beginning in April 1999 (visit 31), each MACS study visit included anthropomorphic measurements. At that time, there were 1952 men under observation, including 849 HIV-infected men and 1103 HIV-uninfected men. Participants for the Lipodystrophy Substudy were recruited between 1999 and 2002. Cases were identified by standardized clinical examinations that were completed semi-annually at each study site, as previously described [9]. HIV-infected men were eligible for recruitment if they had: 1) mild, moderate, or severe fat atrophy involving the face, legs, arms, or buttocks, and 2) mild, moderate, or severe fat hypertrophy involving the breast or abdomen. "Mild" was defined as "only noted after close inspection". "Moderate" was defined as fat changes "noticed by the clinician without specifically looking for them". "Severe" was defined as fat changes "easily noted by a casual observer."

Two control groups were recruited: 1) HIV-infected men without evidence of lipodystrophy by clinical examination and 2) HIV-uninfected healthy men. Controls were matched to cases for age within 5 years and by MACS site. All HIV-infected men were required to have consistently received the same level of antiretroviral treatment (i.e., none, monotherapy or non-HAART combination therapy, or HAART) during the 2 years prior to study entry. HAART was defined according to the US Department of Health and Human Services (DHHS) Kaiser Panel guidelines [10] as previously described[11]. Men with diabetes mellitus or who reported using androgens, anabolic steroids, or other hormonal agents such as megesterol were not eligible for the substudy.

Of the HIV-infected men under follow-up between 1999 and 2002, 281 met criteria for study entry and could be evaluated for exclusion criteria. Of these, 135 were excluded due to use of hormonal agents and 34 due to diabetes, and 35 due to inconsistent antiretroviral therapy level in the 2 years preceding the first clinical evidence of lipodystrophy. Of the remaining 77 men, matched HIV-infected and HIV-uninfected controls could be found for 60, which constituted the recruitment pool for cases.

### Study Procedures

Substudy participants underwent body composition measurements, including anthropometry, CT of the abdomen and thigh, and total body DXA. Body circumferences (waist, hip, thigh), weight, and height were measured using the protocol established in the Third National Health and Nutrition Examination Survey (NHANES III)[12] by trained examiners, as previously described[8]. A wall-mounted stadiometer was used to measure height. Each participant was weighed while wearing minimal clothing or an examination gown. The anthropometric exam was repeated at each subsequent semi-annual MACS study visit.

Quantitative CT was used to measure visceral and subcutaneous adipose tissue. For the abdominal scan, one axial image with 3–10 mm slice thickness was obtained using the space between the fourth and fifth lumbar vertebrae as the origin point. For the thigh scan, one axial image with 3–10 mm slice thickness was acquired using the midpoint of the total femur length as the origin point. Images were sent digitally from each MACS site and were analyzed centrally (Obesity Research Center, Columbia University, New York) using image analysis software (Tomovision Inc., Montreal, Canada). Adipose tissue was identified by selecting the pixels that ranged between -190 and -30 Houndsfield units. The sum of specific tissue pixels multiplied by the individual pixel surface area yielded the respective tissue areas (cm^2^). Adipose tissue areas were calculated for visceral adipose tissue (VAT), subcutaneous adipose tissue (SAT) of the abdomen, and SAT of the thigh. The coefficient of variation was 2–5%.

Whole body dual-energy x-ray absorptiometry (DXA) was undertaken to assess whole body tissue composition (total lean body mass, percent body fat) and regional body composition (trunk fat, extremity fat). Procedures were done using a Lunar Prodigy (GE Medical Systems, Madison, WI) in conjunction with Encore 2002 software at the Pittsburgh site and Hologic 4500A machines with QDA4500A software version 9.03 (Hologic Inc, Waltham, MA) at the other sites.

### Statistical analysis

Categorical demographic variables were compared between the three groups using χ^2 ^testing. Continuous demographic, anthropometric and body composition variables were compared using analysis of covariance (ANCOVA), adjusted for the MACS site and race (white vs. non-white) using PROC GLM in SAS version 9.0 (SAS Institute, Cary, NC)[13]. Since the HIV-infected men in the MACS have a lower mean BMI than the HIV-uninfected men, as previously noted[9,11], and this may have confounded the comparison of regional body composition between the case and the control groups, the data were also adjusted by 1) by MACS site, race, and BMI; or 2) MACS site, race, and lean body mass. Adjusted means were obtained via the LS-means option in PROC GLM, and pairwise comparisons between the groups were made via t-tests using the *pdiff *option in the LS-means statement of PROC GLM.

To determine whether longitudinal changes in anthropomorphic measurements differed between the three groups, multivariable linear mixed effects regression models were implemented. Measurements that were larger than the upper quartile + 1.5 (upper quartile – lower quartile) or smaller than the lower quartile – 1.5 (upper quartile – lower quartile) were considered outliers and were excluded from the analysis. SAS PROC MIXED procedure with a random intercept was used to account for the correlation of repeated measurements. The dependent variables were waist, hip, and thigh circumferences. The independent variables were the study group, BMI at the baseline visit, MACS site, age centered at 50 yrs, and race. P-values < 0.05 were considered significant. Longitudinal changes in anthropometrics for the entire MACS cohort between 1999 and 2003 have been previously reported[11].

## Results

### Substudy Population Characteristics

Eighty-eight men were included from the four MACS sites: 32 HIV-uninfected men, 23 HIV-infected men without clinical lipodystrophy (LIPO-), and 33 HIV-infected men with clinical lipodystrophy (LIPO+). Clinician-generated severity ratings for lipoatrophy were: 10 (30%) mild, 11 (33%) moderate, and 12 (36%) severe. Severity ratings for lipohypertrophy were: 9 (27%) mild, 17 (52%) moderate, and 7 (21%) severe.

Additional file 1 shows the demographic characteristics of the substudy participants. Age was similar among the groups. The HIV-uninfected group had a higher proportion of white participants compared to the two HIV-infected groups. Average BMI and total body fat percentage were lowest in the LIPO+ group, intermediate in the LIPO- group, and highest in the HIV-uninfected group. Lean body mass tended to be lower in the LIPO+ group compared to the other two groups.

The LIPO+ group differed from the LIPO- group at the baseline visit in having a higher proportion of men receiving HAART, and lower current and nadir CD4 cell counts. The mean duration of HAART at the time of enrollment was similar between these two groups.

### Computerized Tomography

Additional file 2 shows CT and DXA measurements, adjusted for: 1) MACS site and race, 2) MACS site, race and BMI, and 3) MACS site, race and lean body mass. VAT was similar among the three groups when adjusted for MACS center and race only. After additional adjustment for BMI, the mean VAT was higher in the LIPO+ group compared to the HIV- (p = 0.07) and LIPO- (p = 0.03). With adjustment for total lean mass, VAT was similar among the groups (p = 0.11), but tended to be higher in LIPO+ than the LIPO- group (p = 0.09).

Amounts of subcutaneous adipose tissue in the abdomen and thigh were lowest in the LIPO+ group, intermediate in the LIPO- group, and highest in the HIV-uninfected men. Adjustment for BMI or lean mass reduced the magnitude of the differences between the groups. After adjustment for BMI, only the differences between the LIPO+ and HIV-uninfected groups remained significant. After adjustment for lean body mass, differences in abdominal SAT were significant between the HIV-uninfected group and the two HIV-infected groups. For thigh SAT, the LIPO+ group was found to have less fat compared to the HIV-infected and LIPO- groups, and thigh SAT tended to be lower in the LIPO- group compared to the HIV-uninfected group (p = 0.06).

### Dual-energy X-ray Absorptiometry

Additional file 2 shows regional body composition measurements as assessed by DXA. Amounts of trunk fat were higher in the HIV-uninfected group compared to the LIPO+ and LIPO- groups. After adjustment for BMI, only the difference between the HIV-uninfected group and the LIPO- group remained significant. After adjustment for lean mass, trunk fat was significantly lower in the HIV-uninfected group than the two HIV-infected groups.

Extremity fat was lowest in the LIPO+ group, intermediate in the LIPO- group, and highest in the HIV-uninfected group. After adjustment for BMI, the differences among the groups became smaller and either non-significant (LIPO+ vs. LIPO- groups) or borderline significant (LIPO- vs. HIV-uninfected group). After adjustment for lean body mass, the amount of extremity fat was significantly lower in the LIPO- group compared to the HIV-uninfected group.

### Anthropomorphic data

Additional file 3 shows the anthropometric data in the three groups. The average waist circumference was highest among HIV-uninfected men; this was significant after adjustment for lean body mass but not for BMI.

Hip circumference was lowest in the LIPO+ group, intermediate in the LIPO- group, and highest in the HIV-uninfected group. After adjustment for BMI, the differences between the HIV-uninfected and each of the HIV-infected groups remained statistically significant. Thigh circumference was lower in both of the HIV-infected groups compared to the HIV-uninfected group, regardless of the adjustment made.

**Figure 1:**
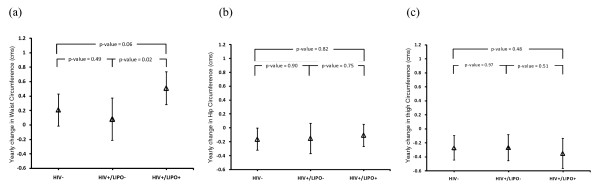
**Average annual changes (2002–2008) in waist (a), hip (b), and thigh (c) circumferences in HIV-uninfected control men (HIV-), HIV-infected men without clinical evidence of lipodystrophy (HIV+LIPO-), and HIV-infected with clinical evidence of lipodystrophy (HIV+LIPO+)**. Error bars represent 95% confidence intervals. Results are adjusted for baseline BMI, age, race, and MACS site.

Figure 1 shows the average rates of change in waist, hip and thigh circumferences over a median of 6 years of follow-up, adjusted for MACS site, age, race, and baseline BMI. Average waist circumference (± standard error) increased significantly in the LIPO+ group (0.51 ± 0.12 cm/year, p < 0.0001), increased borderline significantly in the HIV-uninfected group (0.21 ± 0.11 cm/year, p = 0.07), and did not change in the LIPO- group (0.08 ± 0.15 cm/year, p = 0.59). In contrast, hip circumference did not change significantly in any of the groups, and thigh circumference decreased slightly, but similarly in all groups

## Discussion

In this nested case-control study in the MACS, we used DXA, quantitative CT, and simple anthropometry to compare regional body composition in HIV-infected men with and without clinical evidence of lipodystrophy to HIV-uninfected control subjects. VAT was similar in all 3 groups despite marked differences in BMI, and peripheral lipoatrophy was accentuated in HIV-infected men, regardless of clinical evidence of lipodystrophy, compared to HIV-uninfected men. Finally, over 6 years of follow-up waist circumference increased more rapidly in HIV-infected men who had clinical evidence of lipodystrophy, compared to the HIV-infected men without lipodystrophy and HIV-uninfected men, whereas rate of change in hip and thigh circumference did not differ by group.

While multiple studies have shown that HIV-infected patients with lipodystrophy have more VAT than HIV-infected patients without body composition changes [14,15], relatively few studies have compared VAT in HIV-infected and HIV-uninfected populations [5,16]. One of the difficulties in these comparisons is that BMI tends to be higher in HIV-uninfected populations compared to otherwise similar HIV-infected populations, which may be attributable to differences in energy expenditure[17,18], lipoatrophy, and/or lower lean body mass as a result of chronic HIV infection[19]. In general, VAT tends to be higher in a population with a higher BMI, because of differences in overall adiposity. However, because of lipoatrophy and possibly latent sarcopenia, VAT relative to BMI may be magnified in HIV-infected subjects, posing additional challenges in understanding differences in VAT between HIV-infected and -uninfected subjects.

Cross-sectional studies have taken different approaches to this problem. The FRAM study, the largest such study comparing body composition in men and women with and without HIV infection, reported similar amounts of VAT in 926 HIV-infected and 258 HIV-uninfected subjects[20]. Body mass index, however, was significantly higher in the HIV-uninfected subjects (27.4 ± 5.2 kg/m^2 ^vs. 25.1 ± 4.4 kg/m^2^, p < 0.0001). In some FRAM analyses[5], the differences in body size were accounted for by adjusting for height or lean body mass measured by MRI. In these analyses, differences in VAT by HIV-status were gender-dependent: HIV-infected women without clinical lipoatrophy had more VAT than HIV-infected women with lipoatrophy or HIV-negative controls[6], while HIV-infected men without clinical lipoatrophy tended to have more VAT than HIV-infected men with lipoatrophy, but less than HIV-uninfected controls[5]. Adjustment for body size did not change these relationships. In another cross-sectional study, Joy and colleagues compared regional fat composition, including VAT, in 306 HIV-infected subjects (70% of whom were categorized as having lipodystrophy), and 107 HIV-uninfected controls[16]. To account for the differences in BMI between the two groups, the authors stratified their analysis by BMI category, arguing that, "weight itself may influence the amount of adipose tissue present". In this analysis, both normal weight and overweight HIV-infected men and women had more VAT than gender-matched, HIV-uninfected controls.

In the present study, as in the MACS as a whole [11], HIV-infected men had lower BMIs than HIV-uninfected men. Nevertheless, VAT was similar between the HIV-infected and uninfected groups. To understand the extent to which the similar VAT levels were confounded by the marked differences in body mass, we adjusted for lean body mass as was done in the FRAM study, and this did not alter the lack of difference between the groups. We also adjusted for BMI and found that the differences between HIV-infected men with lipodystrophy and the other two groups were magnified, the largest difference being that between the two HIV-infected groups. In addition, men with clinical evidence of lipodystrophy tended to have higher VAT than the HIV-uninfected control men after adjustment for BMI.

Consistent with the report by Joy et al, which used stratification, our findings suggest that HIV-infected men with clinical lipodystrophy tend to have more VAT for a given BMI than HIV-uninfected men. Because apparent differences in VAT between HIV-infected and -uninfected persons after matching, adjusting, or stratifying on BMI may be inflated, the FRAM investigators did not directly adjust for BMI in their analyses, noting that "BMI is being influenced by the phenomenon being studied: quantity of fat"[5]. Nevertheless, our findings and those of Joy et al have important implications for the clinician. In both HIV-infected and -uninfected populations, increased VAT is associated with cardiovascular risk factors, such as insulin resistance, low HDL cholesterol, and high triglycerides [20-23], and in the general population higher VAT is associated with incident diabetes mellitus and cardiovascular disease[24,25]. Clinicians should be aware that some HIV-infected patients, even at a relatively normal BMI, may be at increased risk of adverse metabolic and cardiovascular outcomes attributable to excess VAT.

Our second major finding was that SAT (thigh or abdomen) and extremity fat were markedly lower in HIV-infected men, with or without clinical lipodystrophy, compared to HIV-uninfected controls. After adjustment for BMI or lean body mass, the magnitude and statistical significance of these differences decreased, with differences in thigh SAT between the HIV-infected men without lipodystrophy and the HIV-uninfected group no longer being statistically significant. However, by DXA, extremity fat in HIV-infected men without lipodystrophy was 20–30% lower than in HIV-uninfected men, regardless of the adjustment. This is consistent with the FRAM study, in which HIV-infected subjects with and without clinical lipoatrophy had lower leg fat than HIV-uninfected subjects [5,6]. Taken together, these results underscore the fact that significant lipoatrophy may be present in HIV-infected persons without clinical evidence of fat wasting and highlight the limitations of using a dichotomous definition of lipoatrophy. Further studies should focus on continuous, objective measures in determining longitudinal changes of body composition in HIV-infected persons and the potential metabolic consequences of mild, subclinical fat wasting.

Few longitudinal data are available on changes in body composition in HIV-infected men with and without clinical lipodystrophy, relative to HIV-uninfected persons. In this study, semi-annual body circumference measurements were available over the 6 years after the substudy visit. HIV-infected men who had clinical evidence of lipodystrophy had a more rapid increase in waist circumference compared to the HIV-infected men without lipodystrophy, and HIV-uninfected men. In contrast, no differences were observed between the groups in the change in hip or thigh circumference over the 6 year interval.

Because measurement of waist circumference does not distinguish between visceral and subcutaneous fat, the more rapid increase in waist circumference in the HIV-infected men with clinical evidence of lipodystrophy could be due to an expansion of either the subcutaneous or visceral fat compartments, or both. Given the severity of lipoatrophy in this group (mean extremity fat 4.5 g), it is possible that some of this increase is due to a reversal of abdominal subcutaneous lipoatrophy. However, if this were the case, more rapid increases in hip and thigh circumference would have also been expected, and these did not occur. Further longitudinal studies, such as the FRAM follow-up study, are required to confirm this finding and understand the extent of change in each of the fat depots in those with a history of body fat abnormalities. Further studies are also needed to understand the factors contributing to the differences in the change of waist circumference in HIV-infected and uninfected patients and whether these are attributable to antiretroviral therapy, increased caloric intake, decreased physical activity, or other factors. In a previous MACS analysis using the entire cohort [8], we found that waist circumference increased more rapidly in HIV-infected men compared to HIV-negative men after adjustment for the effects of antiretroviral therapy, which may suggest a difference in the effect of aging on body composition by HIV-serostatus. Our current findings leave open the possibility that aging-related changes in waist circumference may be accelerated in those with lipodystrophy; this should be further investigated.

The present study had several limitations. First, our cases were defined based on clinical examination alone. In the time since our study was designed, other studies have defined lipodystrophy based on both patient-reported and clinician-observed fat abnormalities[3,5,14] which may reduce bias[26]. Second, the MACS population includes only men and our findings are not generalizable to women. Other studies have shown different patterns of fat distribution in HIV-infected men and women compared to gender-matched control populations [5,6,16]. In addition, our small sample size may have limited our ability to detect small differences between the groups and precluded analyses based on further stratification of the data, such as comparison of BMI categories. Further studies of body composition comparing HIV-infected subjects and HIV-uninfected controls are required, particularly longitudinal studies to assess changes over time.

## Conclusion

Lipoatrophy and lipohypertrophy are common in HIV-infected individuals and are associated with increased cardiometabolic risk and impaired quality of life. Our findings would suggest that even those HIV-infected without clinical evidence of lipoatrophy have reduced subcutaneous and extremity fat compared to their HIV-uninfected peers, highlighting the importance of subclinical lipoatrophy. Our findings would also suggest that abdominal adiposity increases more quickly in HIV-infected men with clinical lipodystophy, compared to those HIV-infected men without lipodsytrophy and HIV-uninfected men. The mechanisms underlying this process and its effects on cardiovascular risk require further investigation.

## Competing interests

TTB has served as a consultant to Abbott Laboratories, EMD Serono, and has received research support from Theratechnologies, Inc, GSK, and Abbott Laboratories. MDW has served as a consultant to Gilead Sciences and Tibotec Pharmaceuticals and has received research support from Tibotec Pharmaceuticals. XX, MJ, JS, FJP, LAK, JBM, and ASD declare that they have no competing interests.

## Authors' contributions

TTB drafted the manuscript and directed the statistical analysis. XX and MJ performed the statistical analysis and helped draft the manuscript. JS helped draft the manuscript and assisted with database management. LAK and FJP participated in the design of the study, assisted with its execution, and assisted with the interpretation of the data. MDW provided administrative and intellectual support for the study. JBM participated in the design of the study, assisted with its execution and provided administrative and intellectual support for the study. ASD conceived of the study design and was responsible for its conduct. All authors read and approved the final manuscript.

## Supplementary Material

Additional file 1 **Supplementary Table 1**. Study population characteristics. Click here for file

Additional file 2 **Supplementary Table 2**. Body composition by computerized tomography (CT) and dual-energy X-ray absorptiometry (DXA) in HIV-uninfected men (HIV-), HIV-infected men without clinical evidence of lipodystrophy (HIV+LIPO-), and HIV-infected with clinical evidence of lipodystrophy (HIV+LIPO+). Click here for file

Additional file 3 **Supplementary Table 3**. Anthropometry in HIV-uninfected control men (HIV-), HIV-infected men without clinical evidence of lipodystrophy (HIV+LIPO-), and HIV-infected with clinical evidence of lipodystrophy (HIV+LIPO+). Click here for file
