# Supplementary material for: Fat distribution and longitudinal anthropometric changes in HIV-infected men with and without clinical evidence of lipodystrophy and HIV-uninfected controls: A substudy of the Multicenter AIDS Cohort Study
Source: AIDS Res Ther. 2009 May 13;6:8. doi: 10.1186/1742-6405-6-8 (PMC2686733; doi:10.1186/1742-6405-6-8)
Supplement: Additional file 1 — Supplementary Table 1. Study population characteristics. [file 1742-6405-6-8-S1.doc]

Supplementary Table 1: Study population characteristics (HIV-:HIV-uninfected men; HIV+LIPO-:HIV-infected men without clinical evidence of lipodystrophy; HIV+LIPO+: HIV-infected with clinical evidence of lipodystrophy)

| **Characteristics** | **HIV-** | **HIV+LIPO-** | **HIV+LIPO+** | **p** | **p for pairwise comparisons** | | |
| --- | --- | --- | --- | --- | --- | --- | --- |
|  |  |  |  |  | **HIV- vs**  **HIV+/LIPO-** | **HIV- v.**  **HIV+/LIPO+** | **HIV+/LIPO- v.**  **HIV+/LIPO+** |
| **Number of participants** | 32 | 23 | 33 | - | - | - | - |
| **Age (years)** | 50.5 (1.2) | 48.4 (1.1) | 50.4 (1.3) | 0.45 | 0.26 | 0.98 | 0.27 |
| **% White** | 96.9% | 78.3% | 72.7% | 0.03 | 0.03 | 0.007 | 0.64 |
| **BMI (kg/m^2^)** | 28.7 (0.9) | 26.8 (1.5) | 23.6 (0.4) | < 0.001 | 0.16 | <0.0001 | 0.02 |
| **Total lean (kg)** | 73.9 (2.6) | 71.3 (2.8) | 65.8 (2.4) | 0.07 | 0.51 | 0.03 | 0.16 |
| **Total Percent fat** | 26.0 (1.0) | 20.3 (1.3) | 17.5 (0.8) | <0.0001 | <0.001 | <0.0001 | 0.08 |
| **CD4 cell count (cell/mm^3^)** | - | 604.3 (54.9) | 534.1 (45.5) | <0.0001 | - | - | <0.0001 |
| **Nadir CD4 cell count (cell/mm^3^)** | - | 307.8 (48.1) | 229.5 (27.0) | <0.0001 | - | - | <0.0001 |
| **Viral load < 400 copies/ml (%)** | - | 66.7% | 50.0% | 0.24 | - | - | 0.24 |
| **Number of participants receiving HAART** | - | 14 (60.1%) | 30 (90.1%) | 0.02 | - | - | 0.02 |
| **Duration of HAART (years)** | - | 5.38 (0.27) | 5.48 (0.40) | 0.84 | - | - | 0.84 |

BMI: Body mass index; HAART: highly active antiretroviral therapy

Values represent mean (standard error), unless otherwise noted
