# Supplementary material for: Fat distribution and longitudinal anthropometric changes in HIV-infected men with and without clinical evidence of lipodystrophy and HIV-uninfected controls: A substudy of the Multicenter AIDS Cohort Study
Source: AIDS Res Ther. 2009 May 13;6:8. doi: 10.1186/1742-6405-6-8 (PMC2686733; doi:10.1186/1742-6405-6-8)
Supplement: Additional file 2 — Supplementary Table 2. Body composition by computerized tomography (CT) and dual-energy X-ray absorptiometry (DXA) in HIV-uninfected men (HIV-), HIV-infected men without clinical evidence of lipodystrophy (HIV+LIPO-), and HIV-infected with clinical evidence of lipodystrophy (HIV+LIPO+). [file 1742-6405-6-8-S2.doc]

Supplementary Table 2: Body composition by computerized tomography (CT) and dual-energy X-ray absorptiometry (DXA) in HIV-uninfected men (HIV-), HIV-infected men without clinical evidence of lipodystrophy (HIV+LIPO-), and HIV-infected with clinical evidence of lipodystrophy (HIV+LIPO+). Values represent means (standard error). *All measurements adjusted for MACS site and race (white vs non-white)

|  | **Additional Adjustment*** | **HIV-** | **HIV+LIPO-** | **HIV+LIPO+** | **p** | **p for pairwise comparisons** | | |
| --- | --- | --- | --- | --- | --- | --- | --- | --- |
|  |  |  |  |  |  | **HIV- v.**  **HIV+/LIPO-** | **HIV- v.**  **HIV+/LIPO+** | **HIV+/LIPO- v.**  **HIV+/LIPO+** |
| **CT measurements (cm^2^)** |  |  |  |  |  |  |  |  |
| **Abdominal VAT** | none | 156.1 (11.9) | 128.1 (13.7) | 149.4 (10.6) | 0.26 | 0.24 | 0.90 | 0.42 |
| **Abdominal VAT** | BMI | 132.7 (11.3) | 128.6 (12.2) | 168.7 (10) | 0.02 | 0.96 | 0.07 | 0.03 |
| **Abdominal VAT** | Lean Mass | 159.1 (10.7) | 139.3 (12.3) | 171.5 (10.7) | 0.11 | 0.41 | 0.661 | 0.09 |
| **Abdominal SAT** | none | 280.3 (21.7) | 186.6 (25) | 103.1 (19.4) | <.0001 | 0.01 | <.0001 | 0.02 |
| **Abdominal SAT** | BMI | 223.8 (15.4) | 189.9 (16.6) | 151.7 (13.7) | 0.006 | 0.27 | 0.004 | 0.17 |
| **Abdominal SAT** | Lean Mass | 285.4 (18.9) | 209.2 (21.8) | 147.1 (18.9) | <.0001 | 0.02 | <.0001 | 0.06 |
| **Thigh SAT** | none | 129.11 (11.84) | 89.63 (13.63) | 33.1 (10.58) | <.0001 | 0.06 | <.0001 | 0.004 |
| **Thigh SAT** | BMI | 105.73 (10.36) | 91.28 (11.2) | 52.56 (9.2) | 0.001 | 0.59 | 0.002 | 0.02 |
| **Thigh SAT** | Lean Mass | 130.53 (11.2) | 98.58 (12.92) | 50.27 (11.2) | <.0001 | 0.13 | <.0001 | 0.009 |
| **DXA Measurements (kg)** |  |  |  |  |  |  |  |  |
| **Trunk fat** | none | 13.62 (0.92) | 9.81 (0.99) | 8.29 (0.87) | <.0001 | 0.009 | <.0001 | 0.44 |
| **Trunk fat** | BMI | 11.66 (0.62) | 9.36 (0.64) | 9.97 (0.58) | 0.02 | 0.02 | 0.12 | 0.74 |
| **Trunk fat** | Lean Mass | 13.86 (0.74) | 10.74 (0.81) | 10.15 (0.75) | 0.0007 | 0.008 | 0.001 | 0.83 |
| **Extremity fat** | none | 9.62 (0.68) | 6.9 (0.73) | 4.47 (0.64) | <.0001 | 0.01 | <.0001 | 0.02 |
| **Extremity fat** | BMI | 8.23 (0.5) | 6.65 (0.52) | 5.66 (0.47) | 0.001 | 0.05 | 0.001 | 0.31 |
| **Extremity fat** | Lean Mass | 9.76 (0.6) | 7.44 (0.65) | 5.54 (0.61) | <.0001 | 0.02 | <.0001 | 0.06 |

VAT: visceral adipose tissue; SAT: subcutaneous adipose tissue.
