# Supplementary material for: Fat distribution and longitudinal anthropometric changes in HIV-infected men with and without clinical evidence of lipodystrophy and HIV-uninfected controls: A substudy of the Multicenter AIDS Cohort Study
Source: AIDS Res Ther. 2009 May 13;6:8. doi: 10.1186/1742-6405-6-8 (PMC2686733; doi:10.1186/1742-6405-6-8)
Supplement: Additional file 3 — Supplementary Table 3. Anthropometry in HIV-uninfected control men (HIV-), HIV-infected men without clinical evidence of lipodystrophy (HIV+LIPO-), and HIV-infected with clinical evidence of lipodystrophy (HIV+LIPO+). [file 1742-6405-6-8-S3.doc]

Supplementary Table 3: Anthropometry in HIV-uninfected control men (HIV-), HIV-infected men without clinical evidence of lipodystrophy (HIV+LIPO-), and HIV-infected with clinical evidence of lipodystrophy (HIV+LIPO+). * All measurements adjusted for MACS site, and race (white vs non-white) and are expressed in centimeters (cm)

|  | **Additional Adjustment*** | **HIV-** | **HIV+LIPO-** | **HIV+LIPO+** | **p-value** | **p for pairwise comparisons** | | |
| --- | --- | --- | --- | --- | --- | --- | --- | --- |
|  |  |  |  |  |  | **HIV- vs**  **HIV+/LIPO-** | **HIV- vs**  **HIV+/LIPO+** | **HIV+/LIPO- vs**  **HIV+/LIPO+** |
| **Waist circumference** | none | 101.04 (2.17) | 93.42 (2.34) | 89.53 (1.93) | <0.001 | 0.04 | <0.001 | 0.39 |
| **Waist circumference** | BMI | 96.33 (1.3) | 92.31 (1.35) | 94.31 (1.17) | 0.08 | 0.06 | 0.50 | 0.49 |
| **Waist circumference** | Lean Mass | 102.19 (1.58) | 96.54 (1.72) | 96.1 (1.6) | <0.01 | 0.03 | 0.01 | 0.98 |
| **Hip circumference** | none | 105.05 (1.57) | 97.8 (1.7) | 91.71 (1.4) | <.0001 | 0.003 | <.0001 | 0.02 |
| **Hip circumference** | BMI | 101.62 (0.93) | 96.98 (0.97) | 95.19 (0.84) | <.0001 | 0.001 | <.0001 | 0.33 |
| **Hip circumference** | Lean Mass | 105.79 (1.05) | 100.12 (1.14) | 96.6 (1.06) | <.0001 | <.0001 | <.0001 | 0.04 |
| **Thigh circumference** | none | 55.91 (1.26) | 50.28 (1.37) | 47.55 (1.12) | <.0001 | 0.006 | <.0001 | 0.26 |
| **Thigh circumference** | BMI | 54.25 (1.15) | 49.89 (1.2) | 49.24 (1.04) | <0.01 | 0.02 | 0.07 | 0.91 |
| **Thigh circumference** | Lean Mass | 56.35 (1.19) | 51.39 (1.29) | 49.88 (1.2) | < 0.001 | 0.009 | 0.0004 | 0.62 |
